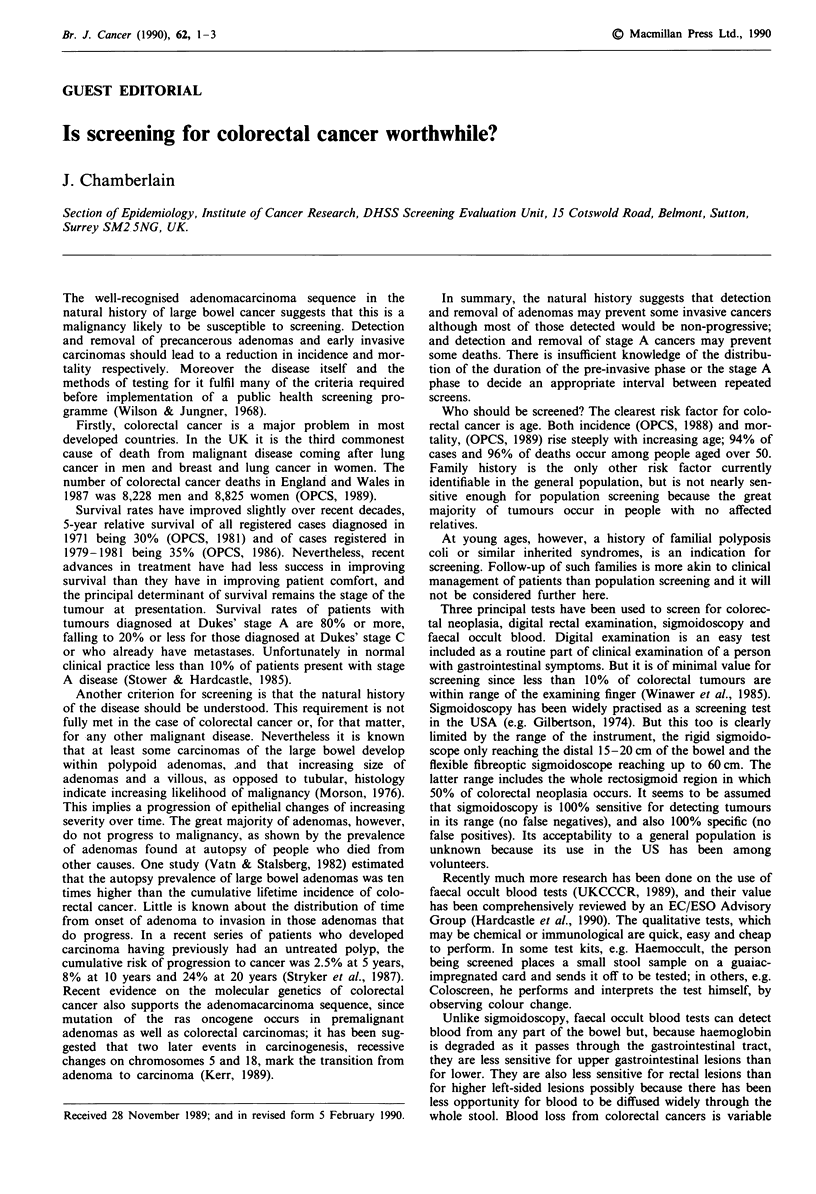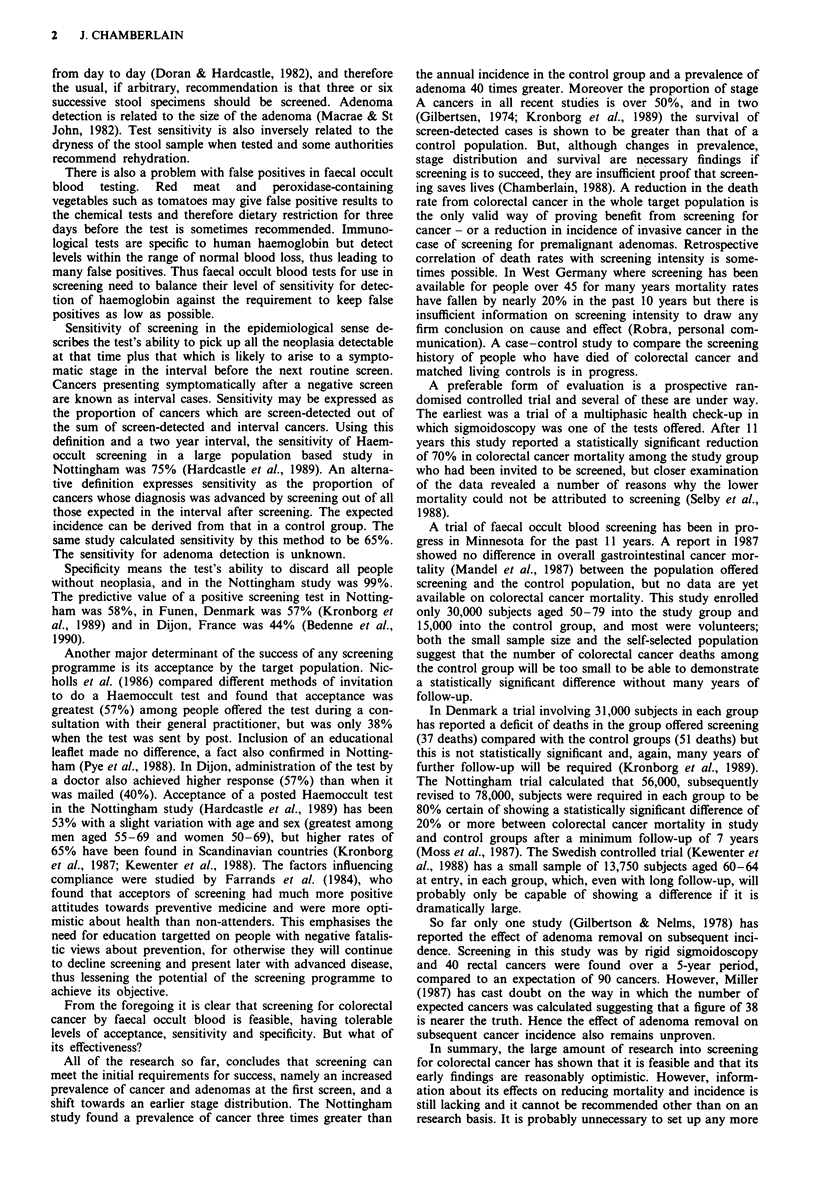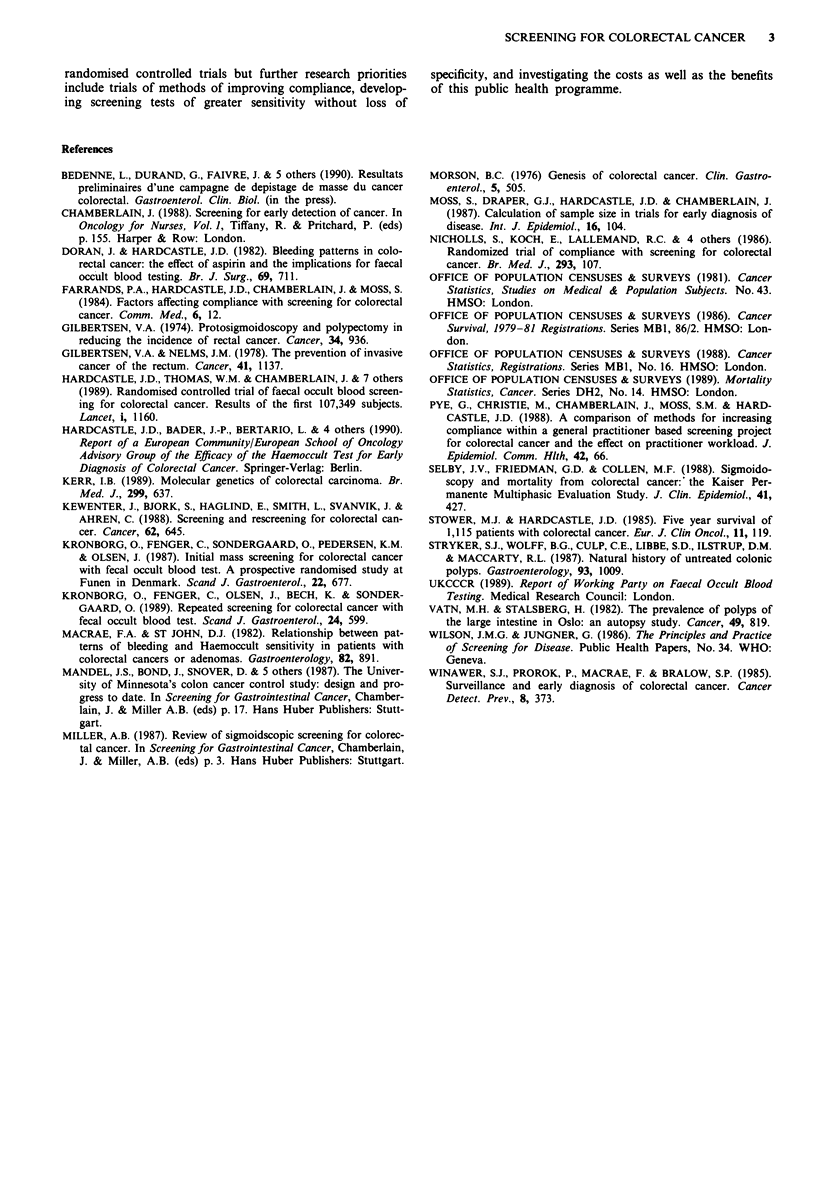# Is screening for colorectal cancer worthwhile?

**DOI:** 10.1038/bjc.1990.216

**Published:** 1990-07

**Authors:** J. Chamberlain

**Affiliations:** Institute of Cancer Research, DHSS Screening Evaluation Unit, Sutton, Surrey, UK.


Br. J. Cancer (1990), 62, 1-3 © Macmillan Press Ltd., 1990

GUEST EDITORIAL

### Is screening for colorectal cancer worthwhile?

J. Chamberlain

*Section of Epidemiology, Institute of Cancer Research, DHSS Screening Evaluation Unit, 15 Cotswold Road, Belmont, Sutton, Surrey SM2 5NG, UK.*

The well-recognised adenomacarcinoma sequence in the natural history of large bowel cancer suggests that this is a malignancy likely to be susceptible to screening. Detection and removal of precancerous adenomas and early invasive carcinomas should lead to a reduction in incidence and mortality respectively. Moreover the disease itself and the methods of testing for it fulfil many of the criteria required before implementation of a public health screening programme (Wilson & Jungner, 1968).

Firstly, colorectal cancer is a major problem in most developed countries. In the UK it is the third commonest cause of death from malignant disease coming after lung cancer in men and breast and lung cancer in women. The number of colorectal cancer deaths in England and Wales in 1987 was 8,228 men and 8,825 women (OPCS, 1989).

Survival rates have improved slightly over recent decades, 5-year relative survival of all registered cases diagnosed in 1971 being 30% (OPCS, 1981) and of cases registered in 1979-1981 being 35% (OPCS, 1986). Nevertheless, recent advances in treatment have had less success in improving survival than they have in improving patient comfort, and the principal determinant of survival remains the stage of the tumour at presentation. Survival rates of patients with tumours diagnosed at Dukes' stage A are 80% or more, falling to 20% or less for those diagnosed at Dukes' stage C or who already have metastases. Unfortunately in normal clinical practice less than 10% of patients present with stage A disease (Stower & Hardcastle, 1985).

Another criterion for screening is that the natural history of the disease should be understood. This requirement is not fully met in the case of colorectal cancer or, for that matter, for any other malignant disease. Nevertheless it is known that at least some carcinomas of the large bowel develop within polypoid adenomas, and that increasing size of adenomas and a villous, as opposed to tubular, histology indicate increasing likelihood of malignancy (Morson, 1976). This implies a progression of epithelial changes of increasing severity over time. The great majority of adenomas, however, do not progress to malignancy, as shown by the prevalence of adenomas found at autopsy of people who died from other causes. One study (Vatn & Stalsberg, 1982) estimated that the autopsy prevalence of large bowel adenomas was ten times higher than the cumulative lifetime incidence of colorectal cancer. Little is known about the distribution of time from onset of adenoma to invasion in those adenomas that do progress. In a recent series of patients who developed carcinoma having previously had an untreated polyp, the cumulative risk of progression to cancer was 2.5% at 5 years, 8% at 10 years and 24% at 20 years (Stryker *et al.*, 1987). Recent evidence on the molecular genetics of colorectal cancer also supports the adenomacarcinoma sequence, since mutation of the ras oncogene occurs in premalignant adenomas as well as colorectal carcinomas; it has been suggested that two later events in carcinogenesis, recessive changes on chromosomes 5 and 18, mark the transition from adenoma to carcinoma (Kerr, 1989).

In summary, the natural history suggests that detection and removal of adenomas may prevent some invasive cancers although most of those detected would be non-progressive; and detection and removal of stage A cancers may prevent some deaths. There is insufficient knowledge of the distribution of the duration of the pre-invasive phase or the stage A phase to decide an appropriate interval between repeated screens.

Who should be screened? The clearest risk factor for colorectal cancer is age. Both incidence (OPCS, 1988) and mortality, (OPCS, 1989) rise steeply with increasing age; 94% of cases and 96% of deaths occur among people aged over 50. Family history is the only other risk factor currently identifiable in the general population, but is not nearly sensitive enough for population screening because the great majority of tumours occur in people with no affected relatives.

At young ages, however, a history of familial polyposis coli or similar inherited syndromes, is an indication for screening. Follow-up of such families is more akin to clinical management of patients than population screening and it will not be considered further here.

Three principal tests have been used to screen for colorectal neoplasia, digital rectal examination, sigmoidoscopy and faecal occult blood. Digital examination is an easy test included as a routine part of clinical examination of a person with gastrointestinal symptoms. But it is of minimal value for screening since less than 10% of colorectal tumours are within range of the examining finger (Winawer *et al.*, 1985). Sigmoidoscopy has been widely practised as a screening test in the USA (e.g. Gilbertson, 1974). But this too is clearly limited by the range of the instrument, the rigid sigmoidoscope only reaching the distal 15-20 cm of the bowel and the flexible fibreoptic sigmoidoscope reaching up to 60 cm. The latter range includes the whole rectosigmoid region in which 50% of colorectal neoplasia occurs. It seems to be assumed that sigmoidoscopy is 100% sensitive for detecting tumours in its range (no false negatives), and also 100% specific (no false positives). Its acceptability to a general population is unknown because its use in the US has been among volunteers.

Recently much more research has been done on the use of faecal occult blood tests (UKCCCR, 1989), and their value has been comprehensively reviewed by an EC/ESO Advisory Group (Hardcastle *et al.*, 1990). The qualitative tests, which may be chemical or immunological are quick, easy and cheap to perform. In some test kits, e.g. Haemoccult, the person being screened places a small stool sample on a guaiac-impregnated card and sends it off to be tested; in others, e.g. Coloscreen, he performs and interprets the test himself, by observing colour change.

Unlike sigmoidoscopy, faecal occult blood tests can detect blood from any part of the bowel but, because haemoglobin is degraded as it passes through the gastrointestinal tract, they are less sensitive for upper gastrointestinal lesions than for lower. They are also less sensitive for rectal lesions than for higher left-sided lesions possibly because there has been less opportunity for blood to be diffused widely through the whole stool. Blood loss from colorectal cancers is variable

Received 28 November 1989; and in revised form 5 February 1990.

from day to day (Doran & Hardcastle, 1982), and therefore the usual, if arbitrary, recommendation is that three or six successive stool specimens should be screened. Adenoma detection is related to the size of the adenoma (Macrae & St John, 1982). Test sensitivity is also inversely related to the dryness of the stool sample when tested and some authorities recommend rehydration.

There is also a problem with false positives in faecal occult blood testing. Red meat and peroxidase-containing vegetables such as tomatoes may give false positive results to the chemical tests and therefore dietary restriction for three days before the test is sometimes recommended. Immunological tests are specific to human haemoglobin but detect levels within the range of normal blood loss, thus leading to many false positives. Thus faecal occult blood tests for use in screening need to balance their level of sensitivity for detection of haemoglobin against the requirement to keep false positives as low as possible.

Sensitivity of screening in the epidemiological sense describes the test's ability to pick up all the neoplasia detectable at that time plus that which is likely to arise to a symptomatic stage in the interval before the next routine screen. Cancers presenting symptomatically after a negative screen are known as interval cases. Sensitivity may be expressed as the proportion of cancers which are screen-detected out of the sum of screen-detected and interval cancers. Using this definition and a two year interval, the sensitivity of Haemoccult screening in a large population based study in Nottingham was 75% (Hardcastle *et al.*, 1989). An alternative definition expresses sensitivity as the proportion of cancers whose diagnosis was advanced by screening out of all those expected in the interval after screening. The expected incidence can be derived from that in a control group. The same study calculated sensitivity by this method to be 65%. The sensitivity for adenoma detection is unknown.

Specificity means the test's ability to discard all people without neoplasia, and in the Nottingham study was 99%. The predictive value of a positive screening test in Nottingham was 58%, in Funen, Denmark was 57% (Kronborg *et al.*, 1989) and in Dijon, France was 44% (Bedenne *et al.*, 1990).

Another major determinant of the success of any screening programme is its acceptance by the target population. Nicholls *et al.* (1986) compared different methods of invitation to do a Haemoccult test and found that acceptance was greatest (57%) among people offered the test during a consultation with their general practitioner, but was only 38% when the test was sent by post. Inclusion of an educational leaflet made no difference, a fact also confirmed in Nottingham (Pye *et al.*, 1988). In Dijon, administration of the test by a doctor also achieved higher response (57%) than when it was mailed (40%). Acceptance of a posted Haemoccult test in the Nottingham study (Hardcastle *et al.*, 1989) has been 53% with a slight variation with age and sex (greatest among men aged 55–69 and women 50–69), but higher rates of 65% have been found in Scandinavian countries (Kronborg *et al.*, 1987; Kewenter *et al.*, 1988). The factors influencing compliance were studied by Farrands *et al.* (1984), who found that acceptors of screening had much more positive attitudes towards preventive medicine and were more optimistic about health than non-attenders. This emphasises the need for education targetted on people with negative fatalistic views about prevention, for otherwise they will continue to decline screening and present later with advanced disease, thus lessening the potential of the screening programme to achieve its objective.

From the foregoing it is clear that screening for colorectal cancer by faecal occult blood is feasible, having tolerable levels of acceptance, sensitivity and specificity. But what of its effectiveness?

All of the research so far, concludes that screening can meet the initial requirements for success, namely an increased prevalence of cancer and adenomas at the first screen, and a shift towards an earlier stage distribution. The Nottingham study found a prevalence of cancer three times greater than the annual incidence in the control group and a prevalence of adenoma 40 times greater. Moreover the proportion of stage A cancers in all recent studies is over 50%, and in two (Gilbertsen, 1974; Kronborg *et al.*, 1989) the survival of screen-detected cases is shown to be greater than that of a control population. But, although changes in prevalence, stage distribution and survival are necessary findings if screening is to succeed, they are insufficient proof that screening saves lives (Chamberlain, 1988). A reduction in the death rate from colorectal cancer in the whole target population is the only valid way of proving benefit from screening for cancer – or a reduction in incidence of invasive cancer in the case of screening for premalignant adenomas. Retrospective correlation of death rates with screening intensity is sometimes possible. In West Germany where screening has been available for people over 45 for many years mortality rates have fallen by nearly 20% in the past 10 years but there is insufficient information on screening intensity to draw any firm conclusion on cause and effect (Robra, personal communication). A case–control study to compare the screening history of people who have died of colorectal cancer and matched living controls is in progress.

A preferable form of evaluation is a prospective randomised controlled trial and several of these are under way. The earliest was a trial of a multiphasic health check-up in which sigmoidoscopy was one of the tests offered. After 11 years this study reported a statistically significant reduction of 70% in colorectal cancer mortality among the study group who had been invited to be screened, but closer examination of the data revealed a number of reasons why the lower mortality could not be attributed to screening (Selby *et al.*, 1988).

A trial of faecal occult blood screening has been in progress in Minnesota for the past 11 years. A report in 1987 showed no difference in overall gastrointestinal cancer mortality (Mandel *et al.*, 1987) between the population offered screening and the control population, but no data are yet available on colorectal cancer mortality. This study enrolled only 30,000 subjects aged 50–79 into the study group and 15,000 into the control group, and most were volunteers; both the small sample size and the self-selected population suggest that the number of colorectal cancer deaths among the control group will be too small to be able to demonstrate a statistically significant difference without many years of follow-up.

In Denmark a trial involving 31,000 subjects in each group has reported a deficit of deaths in the group offered screening (37 deaths) compared with the control groups (51 deaths) but this is not statistically significant and, again, many years of further follow-up will be required (Kronborg *et al.*, 1989). The Nottingham trial calculated that 56,000, subsequently revised to 78,000, subjects were required in each group to be 80% certain of showing a statistically significant difference of 20% or more between colorectal cancer mortality in study and control groups after a minimum follow-up of 7 years (Moss *et al.*, 1987). The Swedish controlled trial (Kewenter *et al.*, 1988) has a small sample of 13,750 subjects aged 60–64 at entry, in each group, which, even with long follow-up, will probably only be capable of showing a difference if it is dramatically large.

So far only one study (Gilbertson & Nelms, 1978) has reported the effect of adenoma removal on subsequent incidence. Screening in this study was by rigid sigmoidoscopy and 40 rectal cancers were found over a 5-year period, compared to an expectation of 90 cancers. However, Miller (1987) has cast doubt on the way in which the number of expected cancers was calculated suggesting that a figure of 38 is nearer the truth. Hence the effect of adenoma removal on subsequent cancer incidence also remains unproven.

In summary, the large amount of research into screening for colorectal cancer has shown that it is feasible and that its early findings are reasonably optimistic. However, information about its effects on reducing mortality and incidence is still lacking and it cannot be recommended other than on an research basis. It is probably unnecessary to set up any more randomised controlled trials but further research priorities include trials of methods of improving compliance, developing screening tests of greater sensitivity without loss of specificity, and investigating the costs as well as the benefits of this public health programme.

### References

BEDENNE, L., DURAND, G., FAIVRE, J. & 5 others (1990). Resultats preliminaires d'une campagne de depistage de masse du cancer colorectal. *Gastroenterol. Clin. Biol.* (in the press).

CHAMBERLAIN, J. (1988). Screening for early detection of cancer. In *Oncology for Nurses, Vol. 1*, Tiffany, R. & Pritchard, P. (eds) p. 155. Harper & Row: London.

DORAN, J. & HARDCASTLE, J.D. (1982). Bleeding patterns in colorectal cancer: the effect of aspirin and the implications for faecal occult blood testing. *Br. J. Surg.*, **69**, 711.

FARRANDS, P.A., HARDCASTLE, J.D., CHAMBERLAIN, J. & MOSS, S. (1984). Factors affecting compliance with screening for colorectal cancer. *Comm. Med.*, **6**, 12.

GILBERTSEN, V.A. (1974). Protosigmoidoscopy and polypectomy in reducing the incidence of rectal cancer. *Cancer*, **34**, 936.

GILBERTSEN, V.A. & NELMS, J.M. (1978). The prevention of invasive cancer of the rectum. *Cancer*, **41**, 1137.

HARDCASTLE, J.D., THOMAS, W.M. & CHAMBERLAIN, J. & 7 others (1989). Randomised controlled trial of faecal occult blood screening for colorectal cancer. Results of the first 107,349 subjects. *Lancet*, **i**, 1160.

HARDCASTLE, J.D., BADER, J.-P., BERTARIO, L. & 4 others (1990). *Report of a European Community/European School of Oncology Advisory Group of the Efficacy of the Haemoccult Test for Early Diagnosis of Colorectal Cancer*. Springer-Verlag: Berlin.

KERR, I.B. (1989). Molecular genetics of colorectal carcinoma. *Br. Med. J.*, **299**, 637.

KEWENTER, J., BJORK, S., HAGLIND, E., SMITH, L., SVANVIK, J. & AHREN, C. (1988). Screening and rescreening for colorectal cancer. *Cancer*, **62**, 645.

KRONBORG, O., FENGER, C., SONDERGAARD, O., PEDERSEN, K.M. & OLSEN, J. (1987). Initial mass screening for colorectal cancer with fecal occult blood test. A prospective randomised study at Funen in Denmark. *Scand J. Gastroenterol.*, **22**, 677.

KRONBORG, O., FENGER, C., OLSEN, J., BECH, K. & SONDERGAARD, O. (1989). Repeated screening for colorectal cancer with fecal occult blood test. *Scand J. Gastroenterol.*, **24**, 599.

MACRAE, F.A. & ST JOHN, D.J. (1982). Relationship between patterns of bleeding and Haemoccult sensitivity in patients with colorectal cancers or adenomas. *Gastroenterology*, **82**, 891.

MANDEL, J.S., BOND, J., SNOVER, D. & 5 others (1987). The University of Minnesota's colon cancer control study: design and progress to date. In *Screening for Gastrointestinal Cancer*, Chamberlain, J. & Miller A.B. (eds) p. 17. Hans Huber Publishers: Stuttgart.

MILLER, A.B. (1987). Review of sigmoidscopic screening for colorectal cancer. In *Screening for Gastrointestinal Cancer*, Chamberlain, J. & Miller, A.B. (eds) p. 3. Hans Huber Publishers: Stuttgart.

MORSON, B.C. (1976) Genesis of colorectal cancer. *Clin. Gastroenterol.*, **5**, 505.

MOSS, S., DRAPER, G.J., HARDCASTLE, J.D. & CHAMBERLAIN, J. (1987). Calculation of sample size in trials for early diagnosis of disease. *Int. J. Epidemiol.*, **16**, 104.

NICHOLLS, S., KOCH, E., LALLEMAND, R.C. & 4 others (1986). Randomized trial of compliance with screening for colorectal cancer. *Br. Med. J.*, **293**, 107.

OFFICE OF POPULATION CENSUSES & SURVEYS (1981). *Cancer Statistics, Studies on Medical & Population Subjects*. No. 43. HMSO: London.

OFFICE OF POPULATION CENSUSES & SURVEYS (1986). *Cancer Survival, 1979–81 Registrations*. Series MB1, 86/2. HMSO: London.

OFFICE OF POPULATION CENSUSES & SURVEYS (1988). *Cancer Statistics, Registrations*. Series MB1, No. 16. HMSO: London.

OFFICE OF POPULATION CENSUSES & SURVEYS (1989). *Mortality Statistics, Cancer*. Series DH2, No. 14. HMSO: London.

PYE, G., CHRISTIE, M., CHAMBERLAIN, J., MOSS, S.M. & HARDCASTLE, J.D. (1988). A comparison of methods for increasing compliance within a general practitioner based screening project for colorectal cancer and the effect on practitioner workload. *J. Epidemiol. Comm. Hlth*, **42**, 66.

SELBY, J.V., FRIEDMAN, G.D. & COLLEN, M.F. (1988). Sigmoidoscopy and mortality from colorectal cancer:' the Kaiser Permanente Multiphasic Evaluation Study. *J. Clin. Epidemiol.*, **41**, 427.

STOWER, M.J. & HARDCASTLE, J.D. (1985). Five year survival of 1,115 patients with colorectal cancer. *Eur. J. Clin Oncol.*, **11**, 119.

STRYKER, S.J., WOLFF, B.G., CULP, C.E., LIBBE, S.D., ILSTRUP, D.M. & MACCARTY, R.L. (1987). Natural history of untreated colonic polyps. *Gastroenterology*, **93**, 1009.

UKCCCR (1989). *Report of Working Party on Faecal Occult Blood Testing*. Medical Research Council: London.

VATN, M.H. & STALSBERG, H. (1982). The prevalence of polyps of the large intestine in Oslo: an autopsy study. *Cancer*, **49**, 819.

WILSON, J.M.G. & JUNGNER, G. (1986). *The Principles and Practice of Screening for Disease*. Public Health Papers, No. 34. WHO: Geneva.

WINAWER, S.J., PROROK, P., MACRAE, F. & BRALOW, S.P. (1985). Surveillance and early diagnosis of colorectal cancer. *Cancer Detect. Prev.*, **8**, 373.